# Severe COVID-19 Lung Infection in Older People and Periodontitis

**DOI:** 10.3390/jcm10020279

**Published:** 2021-01-14

**Authors:** Ruben Aquino-Martinez, Scarlette Hernández-Vigueras

**Affiliations:** 1 Independent Researcher, Rochester, MN 55902, USA; 2 Instituto de Odontoestomatología, Facultad de Medicina, Universidad Austral de Chile, Valdivia 5090000, Chile

**Keywords:** periodontitis, periodontal disease, oral bacteria, *Porphyromonas gingivalis*, LPS, pneumonia, lung, COVID-19, SARS-CoV-2, cellular senescence

## Abstract

Periodontal bacteria dissemination into the lower respiratory tract may create favorable conditions for severe COVID-19 lung infection. Once lung tissues are colonized, cells that survive persistent bacterial infection can undergo permanent damage and accelerated cellular senescence. Consequently, several morphological and functional features of senescent lung cells facilitate SARS-CoV-2 replication. The higher risk for severe SARS-CoV-2 infection, the virus that causes COVID-19, and death in older patients has generated the question whether basic aging mechanisms could be implicated in such susceptibility. Mounting evidence indicates that cellular senescence, a manifestation of aging at the cellular level, contributes to the development of age-related lung pathologies and facilitates respiratory infections. Apparently, a relationship between life-threatening COVID-19 lung infection and pre-existing periodontal disease seems improbable. However, periodontal pathogens can be inoculated during endotracheal intubation and/or aspirated into the lower respiratory tract. This review focuses on how the dissemination of periodontal bacteria into the lungs could aggravate age-related senescent cell accumulation and facilitate more efficient SARS-CoV-2 cell attachment and replication. We also consider how periodontal bacteria-induced premature senescence could influence the course of COVID-19 lung infection. Finally, we highlight the role of saliva as a reservoir for both pathogenic bacteria and SARS-CoV-2. Therefore, the identification of active severe periodontitis can be an opportune and valid clinical parameter for risk stratification of old patients with COVID-19.

## 1. Introduction

In December 2019, severe acute respiratory syndrome coronavirus 2 (SARS-CoV-2) emerged in Wuhan, China. Since then, SARS-CoV-2 has rapidly spread around the world, and today is considered a major global public health threat [1]. Although young adults can be infected with SARS-CoV-2, the risk of developing severe COVID-19 increases with aging. Adults older than 65 years of age represent 80% of hospitalizations and have a higher risk of death compared with younger patients [2]. This higher incidence of severe cases in older patients has generated the question whether basic cellular and molecular mechanisms of aging could be implicated in such susceptibility. On the other hand, the presence of comorbidities increases the risk for severe SARS-CoV-2 infection, leading to poor prognosis. Multiple studies indicate that obesity, hypertension, diabetes, chronic obstructive pulmonary disease (COPD), cardiovascular disease, and cerebrovascular disease are major risk factors for intensive care unit (ICU) admission and death [3,4,5,6]. Consequently, in severe cases of COVID-19 infection, the main causes of death are respiratory failure associated with excessive inflammation (46.91%), followed by septic shock (19.75%), multiorgan failure (16.05%), and cardiac arrest (8.64%) [7]. 

The concept of cellular senescence was formally reported by Hayflick and Moorhead, when they showed that human cells had a limited capacity for replication in vitro [8]. Whereas this finite mitotic lifespan was initially described as an expression of aging at the cellular level, currently, it is recognized that cellular senescence occurs naturally during the aging process of humans and other species [9,10]. Senescent cells gradually accumulate with chronological aging and contribute to the onset and development of chronic age-related pathologies including diabetes, cardiovascular disease, COPD, cerebrovascular inflammation, osteoporosis, and osteoarthritis [11,12,13,14,15,16]. In addition, senescent cells accumulate in adipose tissue of obese humans [17]. Senescent cells negatively impact their local environment by decreasing the regenerative potential of tissues and acting as a potent source of proinflammatory cytokines [10,18,19]. Although cellular senescence is coupled to continuous cell replication and aging, a senescent-like phenotype can be prematurely induced in normal cells by unrelated stressors, which is known as stress-induced premature senescence [20]. Reactive oxygen species (ROS) generated during chronic inflammation, cigarette smoke, and specific Gram-negative bacterial products can accelerate cellular senescence [21,22,23,24]. Therefore, the accumulation of senescent cells in old tissues not only contribute to decreasing tissue repair, but also to increasing local inflammation. 

**Figure 1:**
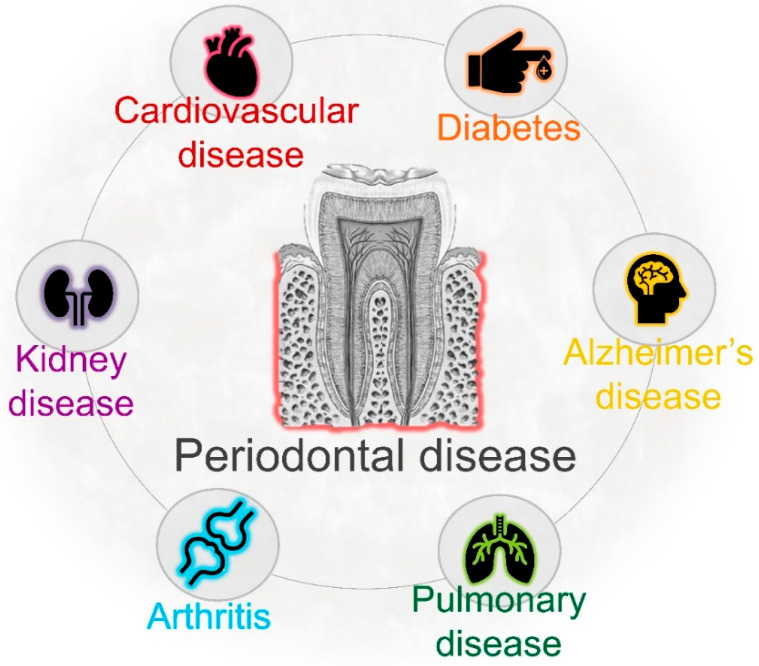
Chronic periodontal disease increases the risk of certain systemic diseases, including respiratory pathologies. Previous studies have demonstrated the association between poor oral health and many systemic conditions.

Periodontal disease, the main cause of tooth loss and masticatory dysfunction in adults, is an infectious condition characterized by the progressive inflammatory destruction of tooth-supporting structures [25,26]. The severity of periodontitis gradually increases with age, showing a rise between 30 and 40 years of age [27]. In the United States, 70% of adults over 65 have periodontal disease [28,29] (Table 1). As consequence of poor oral hygiene, bacteria in dental plaque trigger a local inflammatory reaction, and a complex network of proinflammatory cytokines mediates the recruitment of neutrophils and other inflammatory cells into the site of infection [30,31]. Thus, the main and ultimate cause of periodontal tissue destruction is the host immune defense against pathogenic bacteria. However, periodontal bacteria can also gain access to the bloodstream and disseminate systemically. Once they colonize distant organs, oral bacteria may contribute to initiating disease in those organs [32,33,34,35]. Previous studies have demonstrated an association between severe periodontal disease and hospital-acquired pneumonia, COPD, diabetes, cardiovascular disease, atherosclerosis, cerebrovascular disease, and stroke [36,37,38,39] (Figure 1). This review focuses on the potential role of pre-existing periodontal bacterial infection on the severity of COVID-19 in older people. Specifically, we highlight that translocated *Porphyromonas gingivalis*, a prominent periodontal pathogen with highest prevalence in older adults, may facilitate SARS-CoV-2 replication in lung cells through different mechanisms. We also consider how the inoculation of oral pathogens into the respiratory tract during invasive mechanical ventilation may aggravate SARS-CoV-2-mediated lung inflammation and immune response. 

## 2. Pathogenesis of COVID-19 

Based on their genomic structure, coronaviruses can be divided into four subgroups: α, β, γ, and δ [50]. SARS-CoV-2 is a β-coronavirus, and similar to other respiratory pathogens, can be spread through droplets generated during talking, sneezing, and coughing. In addition, it can be transmitted by “infected” aerosols generated during medical or dental procedures [51,52,53]. The first step for SARS-CoV-2 infection is the binding to angiotensin-converting enzyme 2 (ACE2), a host cell receptor expressed in lung cells and multiple extrapulmonary tissues [54,55]. ACE2 is highly expressed in lung epithelial cells, myocardial cells, the gastrointestinal system, kidney proximal tubule cells, and arterial smooth muscle cells [56]. Furthermore, the oral mucosa plays a crucial role as a portal for SARS-CoV-2 infection, as oral epithelial cells highly express ACE2 [57]. Interestingly, ACE2 expression in the nasal epithelium is age-dependent, with lower expression in children, which may explain the higher COVID-19 prevalence in older individuals [58,59]. Therefore, although different factors are needed for efficient viral infection, human cells with high ACE2 expression are potentially more susceptible to becoming infected with SARS-CoV-2.

After binding specific receptors, viruses manipulate host cells’ resources in order to replicate. Although SARS-CoV-2 is closely related to another coronavirus, it is considered a new β-coronavirus. To better understand a newly identified virus, many mechanisms can be learned from related strains, including invasion and replication properties [60]. For instance, SARS-CoV-2 is able to infect and replicate more efficiently in human lung tissues than SARS-CoV [61]. Some coronaviruses manipulate the mitotic cycle and apoptotic cell death as strategies to promote infection. They can induce cell growth arrest in different cell lines in order to enhance their replication, probably by increasing the availability of deoxynucleotides [62,63,64]. One of the mechanisms used by coronaviruses to induce mitotic arrest is the activation of DNA damage response (DDR) signaling. Xu et al. found that the suppression of ATR (ataxia telangiectasia and Rad3 related), a key modulator of DDR, inhibited viral replication [63]. Although DDR can act as an antiviral mechanism, some viruses can use DDR factors to stimulate replication [65]. Consistent with the concept that coronaviruses can induce cell growth arrest to promote their replication, Bouhaddou et al. recently demonstrated that SARS-CoV-2 causes both DNA damage during the early stage of infection and cell cycle arrest [66]. Therefore, an early cycle inhibitory effect in infected cells may delay their death, and at the same time allow coronaviruses to evade host immune surveillance and facilitate replication [64].

Following viral infection, cells react and secrete specific chemokines that modulate the recruitment of immune cells; this process represents the first line of defense to reduce virus replication and viral spread [67,68]. In contrast, low and dysregulated immune response and reduced levels of antiviral interferons are observed after SARS-CoV-2 infection; however, proinflammatory cytokine secretion remains increased [69,70,71]. High and uncontrolled secretion of proinflammatory cytokines, also known as a “cytokine storm”, can result in severe local tissue injury and also have systemic consequences [68,72]. In agreement with this, a cytokine storm caused as a reaction to SARS-CoV-2 infection is a major cause of direct lung injury, acute respiratory distress syndrome (ARDS), multiple organ failure, and unfavorable prognosis [68,73]. Therefore, lung tissue damage is the consequence not only of the direct damage from virus, but also from ROS-mediated oxidative stress associated with inflammation [74].

## 3. Predictors of Severe Lung COVID-19 Infection

Higher risk of developing severe COVID-19 infection is not the direct consequence of chronological aging itself; it is partially explained by the presence of pre-existing health problems. Indeed, the presence of one or more comorbidities can predict COVID-19 severity in older patients [75,76,77]. Given that severe COVID-19 infection can cause lung damage and dysfunction, it is reasonable to assume that chronic respiratory problems can increase the risk of mortality. Indeed, dyspnea, pneumonia, and COPD are considered the strongest predictive factors for disease severity [78,79]. In addition to the role of pre-existing comorbidities, a dysfunctional immune response has an important role in COVID-19 severity. It has been suggested that the combination of age-related decline of the host immune defense (immunosenescence), chronic low-grade systemic inflammation (inflammaging), and SARS-CoV-2 infection can predispose older patients to increased complications [80]. Along the same line, lymphopenia is also a reliable indicator for disease severity [81].

CT scans and chest radiographic findings, specific cytokines in serum, and the viral load also help to predict COVID-19 severity and potential complications. Given that bilateral lung involvement and pulmonary opacities increase in frequency during the late stage of infection, they are considered hallmarks of SARS-CoV-2 lung infection [82,83,84]. However, after the onset of symptoms (0–2 days), 56% of patients with SARS-CoV-2 infection had normal chest CT scans. In addition, only 28% had bilateral lung involvement, which suggests a limited predictive value during the early stages of infection [84]. On the other hand, during lung infection, immune and nonimmune cells display an excessive secretion of proinflammatory cytokines, including interleukin 6 (IL-6) and tumor necrosis factor-α (TNF-α) [85]. Consistent with this, Del Valle et al. identified that high IL-6 and TNF-α serum levels at the time of hospital admission are strong predictors for disease severity and patient survival [86]. In this inflammatory setting, the hyperactivation of NF-kB has a crucial role in modulating the secretion of proinflammatory mediators and the immune response [85]. Another important parameter relevant to predicting disease severity is the viral load, which in patients with severe infection is around 60 times higher than those with mild disease [87]. Altogether, different parameters can help with the stratification of patients with COVID-19, and the identification of additional clinical factors could increase the predictive capacity of physicians, improving the therapeutic management of patients.

## 4. Periodontal Infection and Systemic Health

There is a general perception that periodontal pathogens and their products exclusively affect the oral cavity; however, periodontal infection can affect extra-oral tissues and contribute to the development of systemic diseases [32,33,34]. Different mechanisms have been proposed to explain how periodontal bacteria could colonize distant organs. One of these mechanisms is bacteremia. During established periodontal inflammation, the gingival epithelial barrier is damaged and ulcerations are produced. Thus, the anatomical closeness between subgingival Gram-negative bacteria and the bloodstream facilitates their systemic dissemination. However, transient bacteremia can also be produced after tooth brushing, flossing, chewing, and dental procedures [88,89,90]. Diverse bacterial species have been identified in blood samples following tooth extraction and tooth brushing, including *Fusobacterium*, *Actinomyces*, and *Prevotella* [90,91]. Remarkably, viable *P. gingivalis* and *A. actinomycetemcomitans*, key microorganisms implicated in periodontal disease pathogenesis, have been identified in human atherosclerotic plaques [92]. Whereas *P. gingivalis* and *A. actinomycetemcomitans* are more commonly identified, other species such as *Tannerella forsythia*, *Fusobacterium nucleatum*, *Eikenella corrodens*, and *Campylobacter rectus* are also found in atheromatous plaques. In fact, in the same atheromatous samples, different bacterial species associated with the etiology of periodontitis can simultaneously be detected [93]. Furthermore, *P. gingivalis* has also been identified in brain tissue of Alzheimer’s disease patients [35]. These studies strongly suggest that under favorable conditions, periodontal pathogens can disseminate and colonize distant organs (Figure 1).

Although different mechanisms have been suggested to explain the translocation of oral bacteria into the respiratory tract, aspiration of saliva seems to play a prominent role. The salivary microbiota is a “conglomerate of bacteria shed from oral surfaces”; it can reflect local changes in supra- and subgingival bacteria, and thus serve as a reservoir of multiple pathogens [94]. Consistent with this concept, it has been recently reported that aspiration of periodontal pathogens may contribute to aggravating SARS-CoV-2 lung infection, in part by aggravating the secretion of key inflammatory cytokines, such as IL-6 [95]. On the other hand, saliva is also a prominent route of COVID-19 transmission. Saliva droplets can contain a high viral load, especially during the early stages of infection [96]. This could contribute to spreading the virus when infected individuals sneeze or cough, but also, saliva droplet inhalation could transport the virus into the lower respiratory tract [97]. However, the relationship between periodontal disease and lung disease does not necessarily require the aspiration of bacteria; periodontal inflammation can also serve as a source of multiple proinflammatory cytokines that can disseminate systemically through the bloodstream, which represents a risk factor for many noncommunicable diseases [98].

Similar to living bacteria, bacterial-derived products and toxins can access the bloodstream. Periodontal Gram-negative bacteria continuously challenge the host immune defense, but they are also a sustained source of Lipopolysaccharide (LPS) and toxins that can reach systemic circulation [99]. Low concentrations of LPS can potentially cause a systemic inflammatory reaction and contribute to abnormal intravascular coagulation and organ dysfunction [100,101,102]. *P. gingivalis*-derived LPS can access brain tissues during life and contribute to neuroinflammation and cognitive impairment [103,104]. In addition, recognized Gram-negative pathogens secrete outer membrane vesicles (OMVs), which provides a sophisticated long-distance delivery system of LPS and other toxins [105]. Periodontal tissues can also act as reservoir of cytokines, such as TNF-α, IL-1α, IL-1β, and IL-6; these proinflammatory mediators may exacerbate pre-existing systemic inflammation [106,107].

## 5. Pre-Existing Periodontal Bacterial Infection and Respiratory Disease

**Figure 2:**
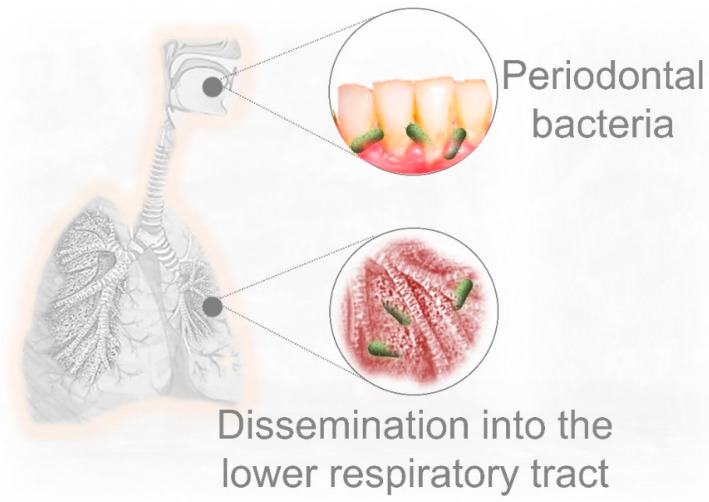
Established periodontal disease is an important source of pathogenic bacteria that can influence the onset and development of lung infection. Periodontal bacteria can disseminate into the lower respiratory system as a consequence of saliva or food aspiration, especially in older individuals, but also inoculation during endotracheal intubation. *P. gingivalis* is a prominent Gram-negative pathogen implicated in the development of periodontal disease, with highest prevalence in elderly people.

Bacteremia and the dissemination of bacterial products are important mechanisms linking periodontal inflammation and respiratory disease. However, the aspiration of oral fluids is a more plausible route by which periodontal pathogens can invade the lower respiratory system. The oral cavity and infected periodontal tissues are potential sources for respiratory pathogens (Figure 2). Thus, the risk of lung infection may increase in those patients with active periodontal infection and poor oral hygiene [48,108,109]. Consistent with the fact that the prevalence of *P. gingivalis* infection increases with age (highest in older adults), this periodontal pathogen has been implicated in the etiology of aspiration pneumonia in elderly people, and also in acute pulmonary infection using murine models [110,111,112]. Whereas periodontal bacteria can be isolated from infected lung samples [108,113], causality of respiratory disease by those microorganisms is difficult to demonstrate. However, oral pathogens can be important additional contributors to aggravate pre-existing lung conditions. Along this line, multiple studies have demonstrated that periodontal treatment has a beneficial effect on lung function by reducing the exacerbation of respiratory diseases. Indeed, significant lower adverse respiratory events, improved lung function, and lower risk of death have been observed in patients with COPD after appropriate periodontal treatment [114,115].

Considering that poor oral hygiene and the aspiration of periodontal bacteria could aggravate COVID-19 lung infection [95], appropriated oral care is a crucial factor to prevent the exacerbation of lung inflammation. Takahashi et al. recently suggested that the aspiration of pathogenic periodontal bacteria exacerbates lung inflammation through the production of cytokines, in particular the elevated production of IL-6. Furthermore, proteases derived from periodontal bacteria could promote SARS-CoV-2 infection [95]. On the other hand, these authors proposed that periodontal bacteria promote viral infection by increasing ACE2 expression due to bacterial products, such as endotoxins. However, when an endotoxin-induced lung inflammation model is used, this last mechanism is controversial because ACE2 activity is reduced by endotoxins (LPS) [116]. In addition, a reduction of ACE2 expression contributes to the pathogenesis of exacerbated lung inflammation, in part by facilitating LPS-induced neutrophil infiltration [116] (see Section 6.2: Periodontal bacteria and exacerbation of lung inflammation in response to SARS-CoV-2). Furthermore, given that the decrease of the viral load in the oral cavity may reduce the risk of SARS-CoV-2 transmission, the use of antiviral mouth rinses could prevent COVID-19 dissemination. In agreement with the prominent role of the oral cavity in SARS-CoV-2 transmission, Carrouel et al. reported that the use of mouth rinses containing povidone-iodine, hydrogen peroxide, chlorhexidine, Citrox, and essential oils, among others, can prevent the spread of SARS-CoV-2. These mouth rinses could decrease the viral load in saliva droplets and decrease the potential risk of dissemination [97]. Consistent with this, the loss of teeth, poor oral hygiene, and higher dental plaque index are strongly associated with COPD exacerbations and severity of dyspnea [117]. Therefore, periodontal inflammation may negatively impact respiratory function, and periodontal therapy could be a preventive approach against respiratory disease exacerbation.

## 6. Could Periodontal Bacteria Dissemination into the Lower Respiratory Tract Contribute to Severe COVID-19 Lung Infection?

The aspiration of oral fluids or food represents a plausible route for the translocation of oral pathogens into the lungs, especially in geriatric patients. In fact, aspiration pneumonia is one of the most common long-term complications of poor oral hygiene in elderly people, and adequate oral health care can decrease the risk of dying from that condition [118,119]. Considering that aspiration pneumonia is often caused by *P. gingivalis* [110,120], and that oral bacteria have been identified in bronchoalveolar lavage samples from COVID-19 patients [121], there are at least three interconnected mechanisms that might facilitate SARS-CoV-2 replication in lung cells as a consequence of pre-existing pulmonary infection by periodontal bacteria: first, *P. gingivalis* LPS-induced senescence; second, exacerbation of local inflammation in response to SARS-CoV-2; third, decreased immune surveillance caused by periodontal bacteria.

### 6.1. LPS-Induced Senescence and SARS-CoV-2 Replication

Cells displaying senescence-like features may facilitate cell entry and promote efficient coronavirus replication. Prolonged exposure to LPS causes accelerated senescence in different cell types, including adipocyte precursors [122], microglial cells [123], and pulmonary epithelial cells [124]. Recent evidence indicates that cells can undergo DNA damage-driven cellular senescence as result of repeated exposure to *P. gingivalis* LPS [125,126]. Although it is recognized that senescent cells contribute to deteriorating their local environment, these dysfunctional cells could also facilitate pathogen infection. Shivshankar et al. demonstrated that senescent cells promote bacterial adhesion to lung cells, eventually resulting in increased susceptibility to bacterial-induced pneumonia in older adults [127]. On the other hand, given that the entry of SARS-CoV-2 in host cells depends on the attachment to specific cell receptors, senescent cells may contribute to facilitating this process.

Vimentin is highly expressed in senescent cells. This filamentous cytoskeletal protein promotes the typical enlarged senescent morphology and plays an essential role in coronavirus cell binding and entry [128,129]. Yu et al. demonstrated that vimentin directly interacts with the spike protein of the coronavirus and that the susceptibility of coronavirus infection is notably reduced by eliminating vimentin activity [129]. Considering that ACE2 expression alone is not sufficient for viral infection, vimentin might act as a surface coreceptor for coronavirus, and likely for SARS-CoV-2 infection as well [129,130,131]. As consequence of this idea, it has been proposed that decreasing vimentin expression could be an attractive strategy for the treatment of COVID-19 infection [131]. Interestingly, Kara et al. recently reported that the strong relationship between severe periodontal disease and COVID-19 could be explained through increased viral attachment and immune response mediated by galectin-3 [132]. Galactin-3 has been identified in the cytoplasm of senescent human fibroblasts, indicating that cells that undergo proliferative arrest lack factors required for the nuclear import of this protein [133]. Furthermore, galectin-3 is highly secreted by senescent mesenchymal stem cells, which could promote the growth of colorectal cancer cells [134]. Consistent with the role of vimentin mentioned above, increased galectin-3 expression by senescent cells could facilitate SARS-CoV-2 attachment and cell entry. Of note, Diaz-Alvarez and Ortega reported that galectin-3 directly binds to invading pathogens and contributes to the immune system reaction against infection [135]. Indeed, galectin-3 is rapidly upregulated in gastric epithelial cells in response to *Helicobacter pylori*, which through the expression of CagA, promotes cellular senescence in human gastric epithelial cells and extra-gastric cells [136,137].

**Figure 3:**
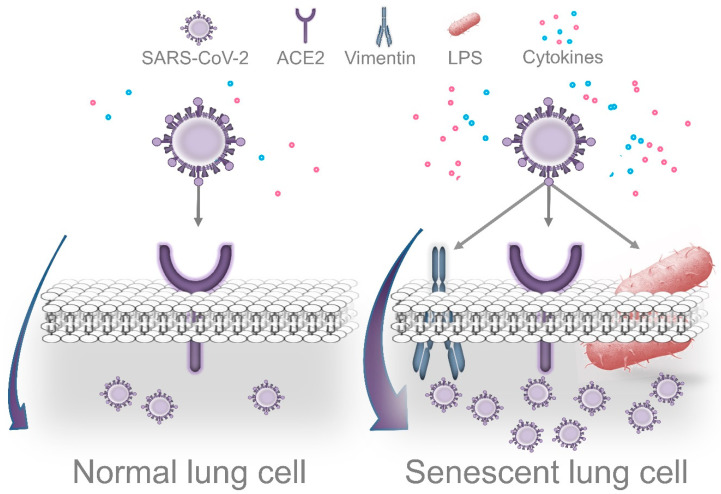
Dissemination of periodontal bacteria into lung tissues may cause Lipopolysaccharide-induced senescence, which facilitates SARS-CoV-2 cell attachment, entry, and replication. Accumulation of senescent cells in lungs is a normal event during the process of aging. Recently, it was demonstrated that cells that survive *P. gingivalis* infection display features of accelerated aging. Thus, periodontal bacteria that colonize lung tissues could aggravate age-related accumulation of senescent cells, which may facilitate more efficient viral replication. Several morphological and functional features of senescent cells could promote this process. In agreement with this concept, the senescence-associated cytoskeletal protein vimentin acts as a coreceptor of coronaviruses’ spike protein, promoting cell binding and entry. Intriguingly, SARS-CoV-2 causes DNA damage, cell growth arrest, cytokine production, and p38 activation during the early stages of infection as a strategy to promote more efficient replication. These are prominent features of cellular senescence. On the other hand, LPS interacts with the SARS-CoV-2 spike protein, which potentiates the inflammatory reaction produced by low concentrations of LPS alone.

Importantly, Bouhaddou et al. demonstrated that SARS-CoV-2 cell infection promotes the production of several cytokines, cell growth arrest, and p38 MAPK activation. These authors also identified that following SARS-CoV-2 infection, an upregulated IL-6 and TNF-α expression is observed, which was inhibited by SB203580 (a p38 inhibitor). Intriguingly, SARS-CoV-2 replication was also decreased by p38 inhibition [66]. P38 MAPK is a key regulatory pathway implicated in cell growth arrest and modulates the expression of most senescence-associated factors. Consequently, p38 inhibition delays the onset of senescence [138,139]. In agreement with the concept that viruses may use senescent cells to enhance replication, Kim et al. found that primary human bronchial epithelial cells that undergo replicative senescence were more susceptible to viral infection and enhanced replication than nonsenescent cells [140]. Therefore, we speculate that age-related accumulation of senescent cells in lung tissues is aggravated by *P. gingivalis* LPS (and other Gram-negative bacteria that colonize lung tissues), which facilitates SARS-CoV-2 cell binding, entry, and more efficient viral replication. However, additional studies are required to validate this idea (Figure 3).

### 6.2. Periodontal Bacteria and Exacerbation of Lung Inflammation in Response to SARS-CoV-2

ACE2 is crucial for SARS-CoV-2 cell binding and replication, but it also plays an essential role in protecting lung tissues from excessive inflammation. Whereas ACE2 mRNA is expressed in practically all human organs, the tissue distribution of ACE2 protein levels seems to be more restricted. Positive staining for ACE2 has been identified in specific sites of certain organs. For instance, in normal lungs, strong immunostaining for ACE2 is observed in alveolar epithelial cells [141]. In agreement with this, Zhao et al. demonstrated that 83% of cells expressing ACE2 in the lung are type II alveolar epithelial cells, suggesting that these cells can have an important role during SARS-CoV-2 infection [142]. In addition to mediating SARS-CoV-2 cell entry, ACE2 acts as an anti-inflammatory and antioxidant factor. Previous studies have demonstrated that ACE2 has a protective function against oxidative stress and severe inflammatory reactions, in part by inhibiting the NF-kB pathway [143,144,145]. Intriguingly, SARS-CoV—and probably SARS-CoV-2—spike protein binding results in ACE2 downregulation in the lungs of mice [146,147]. Along the same line, SARS-CoV-2 activates NF-kB, leading to cytokine secretion, including IL-6 and TNF-α [85]. Therefore, ACE2 expression is reduced after the attachment of the coronavirus spike in host cells, which results in the excessive secretion of key proinflammatory cytokines as a consequence of NF-kB upregulation.

Consistent with this protective role through the inhibition of NF-kB activity, ACE2 prevents both LPS-induced acute respiratory distress syndrome (ARDS) in a murine model and inflammatory injury in pulmonary microvascular endothelial cells [148,149]. Furthermore, ACE2 significantly decrease LPS-promoted IL-6 secretion by 75.6% and IL-1β by 86.7% in lung alveolar epithelial cells in vitro [150]. In addition, ACE2 protects mice from severe acute lung injury and failure induced by acid aspiration [151]. These studies suggest that ACE2 could defend lung tissues from LPS’s detrimental effects. A key finding was recently reported by Petruk et al.: they demonstrated the interaction between the SARS-CoV-2 spike and LPS from *Escherichia coli* and *Pseudomonas aeruginosa*, a Gram-negative bacteria found in the intestines and an opportunistic pathogen implicated in the etiology of ventilator-associated pneumonia (VAP), respectively [152]. More specifically, the SARS-CoV-2 spike protein can interact with lipid A, a component of LPS, and result in significant upregulation of the inflammatory reaction compared with the individual effect of LPS. Moreover, these authors found that the combination of the spike protein and low levels of LPS potentiates NF-kB activation. In addition to decreasing inflammation, ACE2 also could protect lung tissues from LPS-induced oxidative stress. Kim et al. reported that sublethal concentrations of LPS promote the formation of hydrogen peroxide (H_2_O_2_) in a dose-dependent manner in lung alveolar epithelial cells [124]. Consistent with this, acute lung injury induced by LPS is characterized by inflammatory damage and decreased ACE2 expression [153]. On the other hand, Sahni et al. recently proposed that the connection between periodontitis and COVID-19 could be through a common proinflammatory cytokine expression profile; that is, patients with severe symptoms of COVID-19 have increased serum IL-1β, IL-7, IL-10, IL-17, IL-8, TNF-α, and MCP-1 levels, among others [154]. The secretion of many of these cytokines is increased in periodontal tissues of patients with periodontal disease compared to healthy controls. For instance, IL-17 seems to play an important role in exacerbating lung inflammation, and this proinflammatory cytokine can serve as a biomarker of COVID-19 severity [155]. Of note, IL-17 plays an important role in periodontal disease pathogenesis, and it is secreted by senescent alveolar bone osteocytes [125,156]. Altogether, pre-existing Gram-negative bacterial infection and the associated presence of LPS might exacerbate local lung inflammation as result of SARS-CoV-2 spike protein binding by enhancing NF-kB activation.

### 6.3. Impaired Immune Surveillance Caused by Periodontal Bacteria and SARS-CoV-2 Replication

Manipulation of the host immune defense by periodontal bacteria mediates the onset of inflammatory conditions locally, but also at distant organs colonized by those pathogens [157]. Although *P. gingivalis* displays an arsenal of virulence factors, its long-term persistence in invaded tissues depends on its ability to evade immune surveillance. During the early stages of invasion, *P. gingivalis* can delay the recruitment of neutrophils by inhibiting the expression of IL-8, a key chemokine that guides these immune cells into the site of infection. This absence of an IL-8 gradient severely affects local immune defense and promotes the overgrowth of these pathogens. This mechanism is called local chemokine paralysis [158]. However, periodontal pathogens are “inflammophilic” and they depend on inflammation for source nutrients. For that reason, once pathogenic bacteria increase in number and form a biofilm, they “proactively induce inflammation” [159,160].

*P. gingivalis* is also able to invade and survive intracellularly in different cell types, including vascular and epithelial cells in vitro and in vivo [158,161,162]. These pathogens inside epithelial cells may be protected from antibiotics [163]. Interestingly, the ability to decrease immune recognition is not an exclusive feature of *P. gingivalis*. For instance, *T. forsythia*, another pathogen implicated in periodontal disease etiology, can attenuate immune mechanisms of detection, leading to delayed bacterial elimination [164]. Another example is *A. actinomycetemcomitans*, which is recognized by its immunosuppressive properties [165,166]. Considering that with advancing aging, the host immune surveillance is impaired [167,168], these studies suggest that bacterial mechanisms of immune evasion can not only promote their overgrowth, but also facilitate SARS-CoV-2 replication in lung cells.

## 7. Periodontal Status and Ventilator-Associated Pneumonia (VAP)

In patients infected with SARS-CoV-2, progressive respiratory failure is the most common cause for the admission to the ICU [82]. Intubation and invasive ventilation are required in approximately 3.2% of COVID-19 patients in order to provide respiratory support; however, it is also a high-risk procedure with potential complications [169,170,171]. The incidence of VAP ranges from 7% to 70% and represents a major cause of morbidity and mortality in the ICU [172]. VAP is a type of pneumonia caused by “infectious agents not present” at the time of mechanical ventilation, which triggers an inflammatory reaction after 48–72 h following intubation [170,173].

Whereas VAP is a polymicrobial infection, the major group of pathogens isolated from samples of patients with VAP is Gram-negative bacteria [172,174,175,176]. The inoculation of periodontal pathogens, bacteria from the dorsal surface of the tongue, and contaminated upper airway secretions have been directly implicated in the pathogenesis of VAP [177,178]. For example, Okuda et al. found that inoculation of *P. gingivalis* and *T. denticola* into the trachea of mice resulted in increased secretion of cytokines and pneumonia [111]. Furthermore, the proteolytic enzyme gingipain, which is secreted by *P. gingivalis*, mediates lung tissue damage produced during aspiration pneumonia [120]. Moreover, some periodontal pathogens can induce the secretion of proinflammatory cytokines in human lung epithelial cells, as well as in the lower respiratory system using a murine model [179]. These studies suggest that higher risk of oral bacteria inoculation exists when advanced periodontal lesions are present in those patients that require invasive mechanical ventilation. In other words, in older patients with neglected oral care, a greater number of oral bacteria can be attached to the endotracheal tube, translocated into the respiratory tract, and contribute to exacerbated SARS-CoV-2-mediated inflammation as highlighted above.

## 8. Conclusions

Elderly people are more susceptible to experiencing life-threatening SARS-CoV-2 lung infection. Why older patients experience increased COVID-19 severity remains poorly understood. It has been proposed that age-related processes, such as immune defense decline and low-grade systemic inflammation, may play an important role. The periodontal status of older people, on the other hand, has been overlooked. Considering that periodontal bacteria can disseminate into the lower respiratory tract of older people as a result of oral fluid aspiration, or inoculated during invasive mechanical ventilation, it is a plausible hypothesis that translocated Gram-negative periodontal bacteria may cause LPS-induced senescence in lung cells, aggravate age-related senescent cell accumulation, and facilitate SARS-CoV-2 replication. A relationship between established periodontitis and severe SARS-CoV-2 lung infection seems improbable, but *P. gingivalis* LPS-induced accelerated cellular senescence may be an overlooked event that links such conditions. Given that poor oral hygiene and the aspiration of periodontal bacteria could aggravate COVID-19 lung infection, appropriate oral care may be a crucial factor to prevent the exacerbation of lung inflammation. Furthermore, the decrease of the viral load in the oral cavity may reduce the risk of SARS-CoV-2 transmission, and the use of antiviral mouth rinses could prevent COVID-19 dissemination. Although multiple factors are implicated in the pathogenesis of severe COVID-19 lung infection, poor oral hygiene and periodontal infection (“gum disease”) are important parameters to be considered in older people with COVID-19. Therefore, “the added expense of daily oral care is lower than the cost of ignoring it” [180].

## Figures and Tables

**Table 1:** Characteristics of periodontal disease.

	References
Prevalence and severity increased in elderly people	[28,40,41]
Prevalence significantly higher in males	[28,42,43]
Highest prevalence in racial and ethnic minorities	[28,44,45]
Higher severity associated with lower socioeconomic status and education	[28,46,47]
Association with respiratory infection (pneumonia and COPD)	[36,48,49]

## References

[1] Chakraborty I., Maity P. (2020). COVID-19 outbreak: Migration, effects on society, global environment and prevention. Sci. Total Environ..

[2] Mueller A.L., Mcnamara M.S., Sinclair D.A. (2020). Why does COVID-19 disproportionately affect older people?. Aging.

[3] Richardson S., Hirsch J.S., Narasimhan M., Crawford J.M., McGinn T., Davidson K.W., Barnaby D.P., Becker L.B., Chelico J.D., Cohen S.L. (2020). Presenting Characteristics, Comorbidities, and Outcomes Among 5700 Patients Hospitalized With COVID-19 in the New York City Area. JAMA.

[4] Sanyaolu A., Okorie C., Marinkovic A., Patidar R., Younis K., Desai P., Hosein Z., Padda I., Mangat J., Altaf M. (2020). Comorbidity and its Impact on Patients with COVID-19. SN Compr. Clin. Med..

[5] Ji W., Huh K., Kang M., Hong J., Bae G.H., Lee R., Na Y., Choi H., Gong S.Y., Choi Y.H. (2020). Effect of underlying comorbidities on the infection and severity of COVID-19 in Korea: A nationwide case-control study. J. Korean Med. Sci..

[6] Callender L.A., Curran M., Bates S.M., Mairesse M., Weigandt J., Betts C.J. (2020). The Impact of Pre-existing Comorbidities and Therapeutic Interventions on COVID-19. Front. Immunol..

[7] Du Y., Tu L., Zhu P., Mu M., Wang R., Yang P., Wang X., Hu C., Ping R., Hu P. (2020). Clinical features of 85 fatal cases of COVID-19 from Wuhan: A retrospective observational study. Am. J. Respir. Crit. Care Med..

[8] Hayflick L., Moorhead P.S. (1961). The serial cultivation of human diploid cell strains. Exp. Cell Res..

[9] Hayflick L. (1965). The limited in vitro lifetime of human diploid cell strains. Exp. Cell Res..

[10] López-Otín C., Blasco M.A., Partridge L., Serrano M., Kroemer G. (2013). The hallmarks of aging. Cell.

[11] Sone H., Kagawa Y. (2005). Pancreatic beta cell senescence contributes to the pathogenesis of type 2 diabetes in high-fat diet-induced diabetic mice. Diabetologia.

[12] Erusalimsky J.D., Kurz D.J. (2005). Cellular senescence in vivo: Its relevance in ageing and cardiovascular disease. Exp. Gerontol..

[13] Chilosi M., Carloni A., Rossi A., Poletti V. (2013). Premature lung aging and cellular senescence in the pathogenesis of idiopathic pulmonary fibrosis and COPD/emphysema. Transl. Res..

[14] Fulop G.A., Kiss T., Tarantini S., Balasubramanian P., Yabluchanskiy A., Farkas E., Bari F., Ungvari Z., Csiszar A. (2018). Nrf2 deficiency in aged mice exacerbates cellular senescence promoting cerebrovascular inflammation. GeroScience.

[15] Farr J.N., Xu M., Weivoda M.M., Monroe D.G., Fraser D.G., Onken J.L., Negley B.A., Sfeir J.G., Ogrodnik M.B., Hachfeld C.M. (2017). Targeting cellular senescence prevents age-related bone loss in mice. Nat. Med..

[16] McCulloch K., Litherland G.J., Rai T.S. (2017). Cellular senescence in osteoarthritis pathology. Aging Cell.

[17] Palmer A.K., Xu M., Zhu Y., Pirtskhalava T., Weivoda M.M., Hachfeld C.M., Prata L.G., van Dijk T.H., Verkade E., Casaclang-Verzosa G. (2019). Targeting senescent cells alleviates obesity-induced metabolic dysfunction. Aging Cell.

[18] Childs B.G., Durik M., Baker D.J., Van Deursen J.M. (2015). Cellular senescence in aging and age-related disease: From mechanisms to therapy. Nat. Med..

[19] Coppé J.-P., Desprez P.-Y., Krtolica A., Campisi J. (2010). The Senescence-Associated Secretory Phenotype: The Dark Side of Tumor Suppression. Annu. Rev. Pathol. Mech. Dis..

[20] Toussaint O., Medrano E.E., Von Zglinicki T. (2000). Cellular and molecular mechanisms of stress-induced premature senescence (SIPS) of human diploid fibroblasts and melanocytes. Exp. Gerontol..

[21] Feng X., Feng G., Xing J., Shen B., Tan W., Huang D., Lu X., Tao T., Zhang J., Li L. (2014). Repeated lipopolysaccharide stimulation promotes cellular senescence in human dental pulp stem cells (DPSCs). Cell Tissue Res..

[22] Nyunoya T., Monick M.M., Klingelhutz A., Yarovinsky T.O., Cagley J.R., Hunninghake G.W. (2006). Cigarette smoke induces cellular senescence. Am. J. Respir. Cell Mol. Biol..

[23] Blazkova H., Krejcikova K., Moudry P., Frisan T., Hodny Z., Bartek J. (2010). Bacterial intoxication evokes cellular senescence with persistent DNA damage and cytokine signalling. J. Cell. Mol. Med..

[24] Grasso F., Frisan T. (2015). Bacterial Genotoxins: Merging the DNA Damage Response into Infection Biology. Biomolecules.

[25] Page R.C., Schroeder H.E. (1976). Pathogenesis of inflammatory periodontal disease: A summary of current work. Lab. Investig..

[26] Tonetti M.S., Jepsen S., Jin L., Otomo-Corgel J. (2017). Impact of the global burden of periodontal diseases on health, nutrition and wellbeing of mankind: A call for global action. J. Clin. Periodontol..

[27] Kassebaum N.J., Smith A.G.C., Bernabé E., Fleming T.D., Reynolds A.E., Vos T., Murray C.J.L., Marcenes W. (2017). Global, Regional, and National Prevalence, Incidence, and Disability-Adjusted Life Years for Oral Conditions for 195 Countries, 1990–2015: A Systematic Analysis for the Global Burden of Diseases, Injuries, and Risk Factors. J. Dent. Res..

[28] Eke P.I., Dye B.A., Wei L., Thornton-Evans G.O., Genco R.J. (2012). Prevalence of periodontitis in adults in the united states: 2009 and 2010. J. Dent. Res..

[29] Eke P.I., Dye B.A., Wei L., Slade G.D., Thornton-Evans G.O., Borgnakke W.S., Taylor G.W., Page R.C., Beck J.D., Genco R.J. (2015). Update on Prevalence of Periodontitis in Adults in the United States: NHANES 2009 to 2012. J. Periodontol..

[30] Garlet G.P. (2010). Critical reviews in oral biology & medicine: Destructive and protective roles of cytokines in periodontitis: A re-appraisal from host defense and tissue destruction viewpoints. J. Dent. Res..

[31] Pan W., Wang Q., Chen Q. (2019). The cytokine network involved in the host immune response to periodontitis. Int. J. Oral Sci..

[32] Li X., Kolltveit K.M., Tronstad L., Olsen I. (2000). Systemic diseases caused by oral infection. Clin. Microbiol. Rev..

[33] Pizzo G., Guiglia R., Russo L.L., Campisi G. (2010). Dentistry and internal medicine: From the focal infection theory to the periodontal medicine concept. Eur. J. Intern. Med..

[34] Kumar P.S. (2017). From focal sepsis to periodontal medicine: A century of exploring the role of the oral microbiome in systemic disease. J. Physiol..

[35] Dominy S.S., Lynch C., Ermini F., Benedyk M., Marczyk A., Konradi A., Nguyen M., Haditsch U., Raha D., Griffin C. (2019). Porphyromonas gingivalis in Alzheimer’s disease brains: Evidence for disease causation and treatment with small-molecule inhibitors. Sci. Adv..

[36] Scannapieco F.A., Bush R.B., Paju S. (2003). Associations between periodontal disease and risk for nosocomial bacterial pneumonia and chronic obstructive pulmonary disease. A systematic review. Ann. Periodontol..

[37] Beck J., Garcia R., Heiss G., Vokonas P.S., Offenbacher S. (1996). Periodontal Disease and Cardiovascular Disease. J. Periodontol..

[38] Taylor G.W., Borgnakke W.S. (2008). Periodontal disease: Associations with diabetes, glycemic control and complications. Oral Dis..

[39] Sfyroeras G.S., Roussas N., Saleptsis V.G., Argyriou C., Giannoukas A.D. (2012). Association between periodontal disease and stroke. J. Vasc. Surg..

[40] Kassebaum N.J., Bernabé E., Dahiya M., Bhandari B., Murray C.J.L., Marcenes W. (2014). Global burden of severe periodontitis in 1990-2010: A systematic review and meta-regression. J. Dent. Res..

[41] López R., Smith P.C., Göstemeyer G., Schwendicke F. (2017). Ageing, dental caries and periodontal diseases. J. Clin. Periodontol..

[42] Haytac M.C., Ozcelik O., Mariotti A. (2013). Periodontal disease in men. Periodontol. 2000.

[43] Ioannidou E. (2017). The sex and gender intersection in chronic periodontitis. Front. Public Health.

[44] Craig R.G., Yip J.K., Mijares D.Q., Boylan R.J., Haffajee A.D., Socransky S.S. (2003). Destructive periodontal diseases in minority populations. Dent. Clin. N. Am..

[45] Weatherspoon D.J., Borrell L.N., Johnson C.W., Mujahid M.S., Neighbors H.W., Adar S.D. (2016). Racial and ethnic differences in self-reported periodontal disease in the multi-ethnic study of atherosclerosis (MESA). Oral Heal. Prev. Dent..

[46] Borrell L.N., Beck J.D., Heiss G. (2006). Socioeconomic disadvantage and periodontal disease: The dental atherosclerosis risk in communities study. Am. J. Public Health.

[47] Borrell L.N., Crawford N.D. (2012). Socioeconomic position indicators and periodontitis: Examining the evidence. Periodontol. 2000.

[48] Scannapieco F.A. (1999). Role of Oral Bacteria in Respiratory Infection. J. Periodontol..

[49] Barros S.P., Suruki R., Loewy Z.G., Beck J.D., Offenbacher S. (2013). A Cohort Study of the Impact of Tooth Loss and Periodontal Disease on Respiratory Events among COPD Subjects: Modulatory Role of Systemic Biomarkers of Inflammation. PLoS ONE.

[50] Rabi F.A., Al Zoubi M.S., Al-Nasser A.D., Kasasbeh G.A., Salameh D.M. (2020). Sars-cov-2 and coronavirus disease 2019: What we know so far. Pathogens.

[51] Stadnytskyi V., Bax C.E., Bax A., Anfinrud P. (2020). The airborne lifetime of small speech droplets and their potential importance in SARS-CoV-2 transmission. Proc. Natl. Acad. Sci. USA.

[52] Jayaweera M., Perera H., Gunawardana B., Manatunge J. (2020). Transmission of COVID-19 virus by droplets and aerosols: A critical review on the unresolved dichotomy. Environ. Res..

[53] Epstein J.B., Chow K., Mathias R. (2020). Dental procedure aerosols and COVID-19. Lancet Infect. Dis..

[54] Shang J., Wan Y., Luo C., Ye G., Geng Q., Auerbach A., Li F. (2020). Cell entry mechanisms of SARS-CoV-2. Proc. Natl. Acad. Sci. USA.

[55] Gupta A., Madhavan M.V., Sehgal K., Nair N., Mahajan S., Sehrawat T.S., Bikdeli B., Ahluwalia N., Ausiello J.C., Wan E.Y. (2020). Extrapulmonary manifestations of COVID-19. Nat. Med..

[56] Zou X., Chen K., Zou J., Han P., Hao J., Han Z. (2020). Single-cell RNA-seq data analysis on the receptor ACE2 expression reveals the potential risk of different human organs vulnerable to 2019-nCoV infection. Front. Med..

[57] Xu H., Zhong L., Deng J., Peng J., Dan H., Zeng X., Li T., Chen Q. (2020). High expression of ACE2 receptor of 2019-nCoV on the epithelial cells of oral mucosa. Int. J. Oral Sci..

[58] Bunyavanich S., Do A., Vicencio A. (2020). Nasal Gene Expression of Angiotensin-Converting Enzyme 2 in Children and Adults. JAMA J. Am. Med. Assoc..

[59] Patel A.B., Verma A. (2020). Nasal ACE2 Levels and COVID-19 in Children. JAMA J. Am. Med. Assoc..

[60] Belser J.A. (2020). Assessment of SARS-CoV-2 replication in the context of other respiratory viruses. Lancet Respir. Med..

[61] Chu H., Chan J.F.W., Wang Y., Yuen T.T.T., Chai Y., Hou Y., Shuai H., Yang D., Hu B., Huang X. (2020). Comparative replication and immune activation profiles of SARS-CoV-2 and SARS-CoV in human lungs: An ex vivo study with implications for the pathogenesis of COVID-19. Clin. Infect. Dis..

[62] Li F.Q., Tam J.P., Liu D.X. (2007). Cell cycle arrest and apoptosis induced by the coronavirus infectious bronchitis virus in the absence of p53. Virology.

[63] Xu L.H., Huang M., Fang S.G., Liu D.X. (2011). Coronavirus infection induces DNA replication stress partly through interaction of its nonstructural protein 13 with the p125 subunit of DNA polymerase δ. J. Biol. Chem..

[64] Bagga S., Bouchard M.J. (2014). Cell cycle regulation during viral infection. Methods Mol. Biol..

[65] Luftig M.A. (2014). Viruses and the DNA Damage Response: Activation and Antagonism. Annu. Rev. Virol..

[66] Bouhaddou M., Memon D., Meyer B., White K.M., Rezelj V.V., Marrero M.C., Polacco B.J., Melnyk J.E., Ulferts S., Kaake R.M. (2020). The Global Phosphorylation Landscape of SARS-CoV-2 Infection. Cell.

[67] Mogensen T.H., Paludan S.R. (2001). Molecular Pathways in Virus-Induced Cytokine Production. Microbiol. Mol. Biol. Rev..

[68] Ye Q., Wang B., Mao J. (2020). The pathogenesis and treatment of the ‘Cytokine Storm’ in COVID-19. J. Infect..

[69] Blanco-Melo D., Nilsson-Payant B.E., Liu W.C., Uhl S., Hoagland D., Møller R., Jordan T.X., Oishi K., Panis M., Sachs D. (2020). Imbalanced Host Response to SARS-CoV-2 Drives Development of COVID-19. Cell.

[70] Costela-Ruiz V.J., Illescas-Montes R., Puerta-Puerta J.M., Ruiz C., Melguizo-Rodríguez L. (2020). SARS-CoV-2 infection: The role of cytokines in COVID-19 disease. Cytokine Growth Factor Rev..

[71] Catanzaro M., Fagiani F., Racchi M., Corsini E., Govoni S., Lanni C. (2020). Immune response in COVID-19: Addressing a pharmacological challenge by targeting pathways triggered by SARS-CoV-2. Signal. Transduct. Target. Ther..

[72] Tisoncik J.R., Korth M.J., Simmons C.P., Farrar J., Martin T.R., Katze M.G. (2012). Into the Eye of the Cytokine Storm. Microbiol. Mol. Biol. Rev..

[73] Song P., Li W., Xie J., Hou Y., You C. (2020). Cytokine storm induced by SARS-CoV-2. Clin. Chim. Acta.

[74] Tay M.Z., Poh C.M., Rénia L., MacAry P.A., Ng L.F.P. (2020). The trinity of COVID-19: Immunity, inflammation and intervention. Nat. Rev. Immunol..

[75] Atkins J.L., Masoli J.A.H., Delgado J., Pilling L.C., Kuo C.-L., Kuchel G.A., Melzer D. (2020). Preexisting Comorbidities Predicting COVID-19 and Mortality in the UK Biobank Community Cohort. J. Gerontol. Ser. A.

[76] Zhou Y., Yang Q., Chi J., Dong B., Lv W., Shen L., Wang Y. (2020). Comorbidities and the risk of severe or fatal outcomes associated with coronavirus disease 2019: A systematic review and meta-analysis. Int. J. Infect. Dis..

[77] Zhou F., Yu T., Du R., Fan G., Liu Y., Liu Z., Xiang J., Wang Y., Song B., Gu X. (2020). Clinical course and risk factors for mortality of adult inpatients with COVID-19 in Wuhan, China: A retrospective cohort study. Lancet.

[78] Jain V., Yuan J.M. (2020). Predictive symptoms and comorbidities for severe COVID-19 and intensive care unit admission: A systematic review and meta-analysis. Int. J. Public Health.

[79] Wu C., Chen X., Cai Y., Xia J., Zhou X., Xu S., Huang H., Zhang L., Zhou X., Du C. (2020). Risk Factors Associated with Acute Respiratory Distress Syndrome and Death in Patients with Coronavirus Disease 2019 Pneumonia in Wuhan, China. JAMA Intern. Med..

[80] Domingues R., Lippi A., Setz C., Outeiro T.F., Krisko A. (2020). SARS-CoV-2, immunosenescence and inflammaging: Partners in the COVID-19 crime. Aging.

[81] Tan L., Wang Q., Zhang D., Ding J., Huang Q., Tang Y.Q., Wang Q., Miao H. (2020). Lymphopenia predicts disease severity of COVID-19: A descriptive and predictive study. Signal. Transduct. Target. Ther..

[82] Bhatraju P.K., Ghassemieh B.J., Nichols M., Kim R., Jerome K.R., Nalla A.K., Greninger A.L., Pipavath S., Wurfel M.M., Evans L. (2020). COVID-19 in critically ill patients in the Seattle region—Case series. N. Engl. J. Med..

[83] Zhang X., Cai H., Hu J., Lian J., Gu J., Zhang S., Ye C., Lu Y., Jin C., Yu G. (2020). Epidemiological, clinical characteristics of cases of SARS-CoV-2 infection with abnormal imaging findings. Int. J. Infect. Dis..

[84] Bernheim A., Mei X., Huang M., Yang Y., Fayad Z.A., Zhang N., Diao K., Lin B., Zhu X., Li K. (2020). Chest CT findings in coronavirus disease 2019 (COVID-19): Relationship to duration of infection. Radiology.

[85] Hirano T., Murakami M. (2020). COVID-19: A New Virus, but a Familiar Receptor and Cytokine Release Syndrome. Immunity.

[86] Del Valle D.M., Kim-Schulze S., Huang H.H., Beckmann N.D., Nirenberg S., Wang B., Lavin Y., Swartz T.H., Madduri D., Stock A. (2020). An inflammatory cytokine signature predicts COVID-19 severity and survival. Nat. Med..

[87] Liu Y., Yan L.M., Wan L., Xiang T.X., Le A., Liu J.M., Peiris M., Poon L.L.M., Zhang W. (2020). Viral dynamics in mild and severe cases of COVID-19. Lancet Infect. Dis..

[88] Forner L., Larsen T., Kilian M., Holmstrup P. (2006). Incidence of bacteremia after chewing, tooth brushing and scaling in individuals with periodontal inflammation. J. Clin. Periodontol..

[89] Kinane D.F., Riggio M.P., Walker K.F., MacKenzie D., Shearer B. (2005). Bacteraemia following periodontal procedures. J. Clin. Periodontol..

[90] Lockhart P.B., Brennan M.T., Sasser H.C., Fox P.C., Paster B.J., Bahrani-Mougeot F.K. (2008). Bacteremia associated with toothbrushing and dental extraction. Circulation.

[91] Bahrani-Mougeot F.K., Paster B.J., Coleman S., Ashar J., Barbuto S., Lockhart P.B. (2008). Diverse and novel oral bacterial species in blood following dental procedures. J. Clin. Microbiol..

[92] Kozarov E.V., Dorn B.R., Shelburne C.E., Dunn W.A., Progulske-Fox A. (2005). Human atherosclerotic plaque contains viable invasive Actinobacillus actinomycetemcomitans and Porphyromonas gingivalis. Arterioscler. Thromb. Vasc. Biol..

[93] Figuero E., Sánchez-Beltrán M., Cuesta-Frechoso S., Tejerina J.M., del Castro J.A., Gutiérrez J.M., Herrera D., Sanz M. (2011). Detection of Periodontal Bacteria in Atheromatous Plaque by Nested Polymerase Chain Reaction. J. Periodontol..

[94] Belstrøm D. (2020). The salivary microbiota in health and disease. J. Oral Microbiol..

[95] Takahashi Y., Watanabe N., Kamio N., Kobayashi R., Iinuma T., Imai K. (2020). Aspiration of periodontopathic bacteria due to poor oral hygiene potentially contributes to the aggravation of COVID-19. J. Oral Sci..

[96] Yoon J.G., Yoon J., Song J.Y., Yoon S.Y., Lim C.S., Seong H., Noh J.Y., Cheong H.J., Kim W.J. (2020). Clinical significance of a high SARS-CoV-2 viral load in the Saliva. J. Korean Med. Sci..

[97] Carrouel F., Gonçalves L.S., Conte M.P., Campus G., Fisher J., Fraticelli L., Gadea-Deschamps E., Ottolenghi L., Bourgeois D. (2020). Antiviral Activity of Reagents in Mouth Rinses against SARS-CoV-2. J. Dent. Res..

[98] Bourgeois D., Inquimbert C., Ottolenghi L., Carrouel F. (2019). Periodontal pathogens as risk factors of cardiovascular diseases, diabetes, rheumatoid arthritis, cancer, and chronic obstructive pulmonary disease—Is there cause for consideration?. Microorganisms.

[99] Page R.C. (1998). The pathobiology of periodontal diseases may affect systemic diseases: Inversion of a paradigm. Ann. Periodontol..

[100] Inoue K.I., Takano H., Shimada A., Yanagisawa R., Sakurai M., Yoshino S., Sato H., Yoshikawa T. (2005). Urinary trypsin inhibitor protects against systemic inflammation induced by lipopolysaccharide. Mol. Pharmacol..

[101] Slofstra S.H., ten Cate H., Spek C.A. (2006). Low dose endotoxin priming is accountable for coagulation abnormalities and organ damage observed in the Shwartzman reaction. A comparison between a single-dose endotoxemia model and a double-hit endotoxin-induced Shwartzman reaction. Thromb. J..

[102] Levi M., Keller T.T., Van Gorp E., Ten Cate H. (2003). Infection and inflammation and the coagulation system. Cardiovasc. Res..

[103] Poole S., Singhrao S.K., Kesavalu L., Curtis M.A., Crean S.J. (2013). Determining the presence of periodontopathic virulence factors in short-term postmortem Alzheimer’s disease brain tissue. J. Alzheimer’s Dis..

[104] Zhang J., Yu C., Zhang X., Chen H., Dong J., Lu W., Song Z., Zhou W. (2018). Porphyromonas gingivalis lipopolysaccharide induces cognitive dysfunction, mediated by neuronal inflammation via activation of the TLR4 signaling pathway in C57BL/6 mice. J. Neuroinflammation.

[105] Bonnington K.E., Kuehn M.J. (2014). Protein selection and export via outer membrane vesicles. Biochim. Biophys. Acta Mol. Cell Res..

[106] Reis C., Da Costa A.V., Guimarães J.T., Tuna D., Braga A.C., Pacheco J.J., Arosa F.A., Salazar F., Cardoso E.M. (2014). Clinical improvement following therapy for periodontitis: Association with a decrease in IL-1 and IL-6. Exp. Ther. Med..

[107] Konkel J.E., O’Boyle C., Krishnan S. (2019). Distal consequences of oral inflammation. Front. Immunol..

[108] Mojon P. (2002). Oral health and respiratory infection. J. Can. Dent. Assoc..

[109] Scannapieco F.A., Ho A.W. (2001). Potential Associations Between Chronic Respiratory Disease and Periodontal Disease: Analysis of National Health and Nutrition Examination Survey III. J. Periodontol..

[110] Savitt E.D., Kent R.L. (1991). Distribution of Actinobacillus actinomycetemcomitans and Porphyromonas gingivalis by Subject Age. J. Periodontol..

[111] Okuda K., Kimizuka R., Abe S., Kato T., Ishihara K. (2005). Involvement of Periodontopathic Anaerobes in Aspiration Pneumonia. J. Periodontol..

[112] Hajishengallis G., Wang M., Bagby G.J., Nelson S. (2008). Importance of TLR2 in Early Innate Immune Response to Acute Pulmonary Infection with Porphyromonas gingivalis in Mice. J. Immunol..

[113] Caldas R.R., Le Gall F., Revert K., Rault G., Virmaux M., Gouriou S., Héry-Arnaud G., Barbier G., Boisramé S. (2015). Pseudomonas aeruginosa and periodontal pathogens in the oral cavity and lungs of cystic fibrosis patients: A case-control study. J. Clin. Microbiol..

[114] Shen T.C., Chang P.Y., Lin C.L., Chen C.H., Tu C.Y., Hsia T.C., Shih C.M., Hsu W.H., Sung F.C., Kao C.H. (2016). Periodontal Treatment Reduces Risk of Adverse Respiratory Events in Patients with Chronic Obstructive Pulmonary Disease. Medicine.

[115] Zhou X., Han J., Liu Z., Song Y., Wang Z., Sun Z. (2014). Effects of periodontal treatment on lung function and exacerbation frequency in patients with chronic obstructive pulmonary disease and chronic periodontitis: A 2-year pilot randomized controlled trial. J. Clin. Periodontol..

[116] Sodhi C.P., Wohlford-Lenane C., Yamaguchi Y., Prindle T., Fulton W.B., Wang S., McCray P.B., Chappell M., Hackam D.J., Jia H. (2018). Attenuation of pulmonary ACE2 activity impairs inactivation of des-arg9 bradykinin/BKB1R axis and facilitates LPS-induced neutrophil infiltration. Am. J. Physiol. Lung Cell. Mol. Physiol..

[117] Liu Z., Zhang W., Zhang J., Zhou X., Zhang L., Song Y., Wang Z. (2012). Oral hygiene, periodontal health and chronic obstructive pulmonary disease exacerbations. J. Clin. Periodontol..

[118] Van Der Maarel-Wierink C.D., Vanobbergen J.N.O., Bronkhorst E.M., Schols J.M.G.A., De Baat C. (2013). Oral health care and aspiration pneumonia in frail older people: A systematic literature review. Gerodontology.

[119] Terpenning M. (2005). Geriatric oral health and pneumonia risk. Clin. Infect. Dis..

[120] Benedyk M., Mydel P.M., Delaleu N., Płaza K., Gawron K., Milewska A., Maresz K., Koziel J., Pyrc K., Potempa J. (2016). Gingipains: Critical Factors in the Development of Aspiration Pneumonia Caused by Porphyromonas gingivalis. J. Innate Immun..

[121] Shen Z., Xiao Y., Kang L., Ma W., Shi L., Zhang L., Zhou Z., Yang J., Zhong J., Yang D. (2020). Genomic diversity of SARS-CoV-2 in Coronavirus Disease 2019 patients. Clin. Infect. Dis..

[122] Zhao M., Chen X. (2015). Effect of lipopolysaccharides on adipogenic potential and premature senescence of adipocyte progenitors. Am. J. Physiol. Endocrinol. Metab..

[123] Yu H.M., Zhao Y.M., Luo X.G., Feng Y., Ren Y., Shang H., He Z.Y., Luo X.M., Chen S.D., Wang X.Y. (2012). Repeated lipopolysaccharide stimulation induces cellular senescence in BV2 cells. Neuroimmunomodulation.

[124] Kim C.O., Huh A.J., Han S.H., Kim J.M. (2012). Analysis of cellular senescence induced by lipopolysaccharide in pulmonary alveolar epithelial cells. Arch. Gerontol. Geriatr..

[125] Aquino-Martinez R., Rowsey J.L., Fraser D.G., Eckhardt B.A., Khosla S., Farr J.N., Monroe D.G. (2020). LPS-induced premature osteocyte senescence: Implications in inflammatory alveolar bone loss and periodontal disease pathogenesis. Bone.

[126] Aquino-Martinez R., Khosla S., Farr J.N., Monroe D.G. (2020). Periodontal disease and senescent cells: New players for an old oral health problem?. Int. J. Mol. Sci..

[127] Shivshankar P., Boyd A.R., Le Saux C.J., Yeh I.T., Orihuela C.J. (2011). Cellular senescence increases expression of bacterial ligands in the lungs and is positively correlated with increased susceptibility to pneumococcal pneumonia. Aging Cell.

[128] Nishio K., Inoue A., Qiao S., Kondo H., Mimura A. (2001). Senescence and cytoskeleton: Overproduction of vimentin induces senescent-like morphology in human fibroblasts. Histochem. Cell Biol..

[129] Yu Y.T.C., Chien S.C., Chen I.Y., Lai C.T., Tsay Y.G., Chang S.C., Chang M.F. (2016). Surface vimentin is critical for the cell entry of SARS-CoV. J. Biomed. Sci..

[130] Ramos I., Stamatakis K., Oeste C.L., Pérez-Sala D. (2020). Vimentin as a multifaceted player and potential therapeutic target in viral infections. Int. J. Mol. Sci..

[131] Li Z., Paulin D., Lacolley P., Coletti D., Agbulut O. (2020). Vimentin as a target for the treatment of COVID-19. BMJ Open Respir. Res..

[132] Kara C., Çelen K., Dede F.Ö., Gökmenoğlu C., Kara N.B. (2020). Is periodontal disease a risk factor for developing severe Covid-19 infection? The potential role of Galectin-3. Exp. Biol. Med..

[133] Openo K.P., Kadrofske M.M., Patterson R.J., Wang J.L. (2000). Galectin-3 expression and subcellular localization in senescent human fibroblasts. Exp. Cell Res..

[134] Li Y., Xu X., Wang L., Liu G., Li Y., Wu X., Jing Y., Li H., Wang G. (2015). Senescent mesenchymal stem cells promote colorectal cancer cells growth via galectin-3 expression. Cell Biosci..

[135] Díaz-Alvarez L., Ortega E. (2017). The Many Roles of Galectin-3, a Multifaceted Molecule, in Innate Immune Responses against Pathogens. Mediat. Inflamm..

[136] Lewinska A., Wnuk M. (2017). Helicobacter pylori-induced premature senescence of extragastric cells may contribute to chronic skin diseases. Biogerontology.

[137] Fowler M., Thomas R.J., Atherton J., Roberts I.S., High N.J. (2006). Galectin-3 binds to Helicobacter pylori O-antigen: It is upregulated and rapidly secreted by gastric epithelial cells in response to H. pylori adhesion. Cell. Microbiol..

[138] Freund A., Patil C.K., Campisi J. (2011). P38MAPK is a novel DNA damage response-independent regulator of the senescence-associated secretory phenotype. EMBO J..

[139] Iwasa H., Han J., Ishikawa F. (2003). Mitogen-activated protein kinase p38 defines the common senescence-signalling pathway. Genes Cells.

[140] Kim J.A., Seong R.K., Shin O.S. (2016). Enhanced viral replication by cellular replicative senescence. Immune Netw..

[141] Hamming I., Timens W., Bulthuis M.L.C., Lely A.T., Navis G.J., van Goor H. (2004). Tissue distribution of ACE2 protein, the functional receptor for SARS coronavirus. A first step in understanding SARS pathogenesis. J. Pathol..

[142] Zhao Y., Zhao Z., Wang Y., Zhou Y., Ma Y., Zuo W. (2020). Single-Cell RNA Expression Profiling of ACE2, the Receptor of SARS-CoV-2. Am. J. Respir. Crit. Care Med..

[143] Calò L.A., Rigato M., Bertoldi G. (2020). ACE2/Angiotensin 1-7 protective anti-inflammatory and antioxidant role in hyperoxic lung injury: Support from studies in Bartter’s and Gitelman’s syndromes. QJM.

[144] Meng Y., Yu C.H., Li W., Li T., Luo W., Huang S., Wu P.S., Cai S.X., Li X. (2014). Angiotensin-converting enzyme 2/angiotensin-(1-7)/mas axis protects against lung fibrosis by inhibiting the MAPK/NF-κB pathway. Am. J. Respir. Cell Mol. Biol..

[145] Fang Y., Gao F., Liu Z. (2019). Angiotensin-converting enzyme 2 attenuates inflammatory response and oxidative stress in hyperoxic lung injury by regulating NF-jB and Nrf2 pathways. QJM.

[146] Kuba K., Imai Y., Rao S., Gao H., Guo F., Guan B., Huan Y., Yang P., Zhang Y., Deng W. (2005). A crucial role of angiotensin converting enzyme 2 (ACE2) in SARS coronavirus-induced lung injury. Nat. Med..

[147] Zhang H., Penninger J.M., Li Y., Zhong N., Slutsky A.S. (2020). Angiotensin-converting enzyme 2 (ACE2) as a SARS-CoV-2 receptor: Molecular mechanisms and potential therapeutic target. Intensive Care Med..

[148] Li Y., Cao Y., Zeng Z., Liang M., Xue Y., Xi C., Zhou M., Jiang W. (2015). Angiotensin-converting enzyme 2/angiotensin-(1-7)/Mas axis prevents lipopolysaccharide-induced apoptosis of pulmonary microvascular endothelial cells by inhibiting JNK/NF-κB pathways. Sci. Rep..

[149] Li Y., Zeng Z., Cao Y., Liu Y., Ping F., Liang M., Xue Y., Xi C., Zhou M., Jiang W. (2016). Angiotensin-converting enzyme 2 prevents lipopolysaccharide-induced rat acute lung injury via suppressing the ERK1/2 and NF-κB signaling pathways. Sci. Rep..

[150] Wong M.H., Chapin O.C., Johnson M.D. (2012). LPS-stimulated cytokine production in Type I cells is modulated by the renin-angiotensin system. Am. J. Respir. Cell Mol. Biol..

[151] Imai Y., Kuba K., Rao S., Huan Y., Guo F., Guan B., Yang P., Sarao R., Wada T., Leong-Poi H. (2005). Angiotensin-converting enzyme 2 protects from severe acute lung failure. Nature.

[152] Petruk G., Puthia M., Petrlova J., Strömdahl A.-C., Kjellström S., Schmidtchen A. (2020). SARS-CoV-2 Spike protein binds to bacterial lipopolysaccharide and boosts proinflammatory activity. bioRxiv.

[153] Ye R., Liu Z. (2020). ACE2 exhibits protective effects against LPS-induced acute lung injury in mice by inhibiting the LPS-TLR4 pathway. Exp. Mol. Pathol..

[154] Sahni V., Gupta S. (2020). COVID-19 & Periodontitis: The cytokine connection. Med. Hypotheses.

[155] Pacha O., Sallman M.A., Evans S.E. (2020). COVID-19: A case for inhibiting IL-17?. Nat. Rev. Immunol..

[156] Takahashi K., Azuma T., Motohira H., Kinane D.F., Kitetsu S. (2005). The potential role of interleukin-17 in the immunopathology of periodontal disease. J. Clin. Periodontol..

[157] Hajishengallis G. (2015). Periodontitis: From microbial immune subversion to systemic inflammation. Nat. Rev. Immunol..

[158] Darveau R.P., Belton C.M., Reife R.A., Lamont R.J. (1998). Local Chemokine Paralysis, a Novel Pathogenic Mechanism for Porphyromonas gingivalis. Infect. Immun..

[159] Hajishengallis G. (2014). The inflammophilic character of the periodontitis-associated microbiota. Mol. Oral Microbiol..

[160] Hajishengallis G., Lamont R.J. (2014). Breaking bad: Manipulation of the host response by Porphyromonas gingivalis. Eur. J. Immunol..

[161] Li L., Michel R., Cohen J., DeCarlo A., Kozarov E. (2008). Intracellular survival and vascular cell-to-cell transmission of Porphyromonas gingivalis. BMC Microbiol..

[162] Olsen I., Progulske-Fox A. (2015). Invasion of Porphyromonas gingivalis strains into vascular cells and tissue. J. Oral Microbiol..

[163] Eick S., Pfister W. (2004). Efficacy of Antibiotics Against Periodontopathogenic Bacteria Within Epithelial Cells: An In Vitro Study. J. Periodontol..

[164] Sekot G., Posch G., Messner P., Matejka M., Rausch-Fan X., Andrukhov O., Schäffer C. (2011). Potential of the tannerella forsythia S-layer to delay the immune response. J. Dent. Res..

[165] Shenker B.J., McKay T., Datar S., Miller M., Chowhan R., Demuth D. (1999). Actinobacillus actinomycetemcomitans immunosuppressive protein is a member of the family of cytolethal distending toxins capable of causing a G2 arrest in human T cells. J. Immunol..

[166] Rabie G., Lally E.T., Shenker B.J. (1988). Immunosuppressive properties of Actinobacillus actinomycetemcomitants leukotoxin. Infect. Immun..

[167] Muñoz-Espín D., Serrano M. (2014). Cellular senescence: From physiology to pathology. Nat. Rev. Mol. Cell Biol..

[168] Ovadya Y., Landsberger T., Leins H., Vadai E., Gal H., Biran A., Yosef R., Sagiv A., Agrawal A., Shapira A. (2018). Impaired immune surveillance accelerates accumulation of senescent cells and aging. Nat. Commun..

[169] Goyal P., Choi J.J., Pinheiro L.C., Schenck E.J., Chen R., Jabri A., Satlin M.J., Campion T.R., Nahid M., Ringel J.B. (2020). Clinical characteristics of COVID-19 in New York City. N. Engl. J. Med..

[170] Carter C., Osborn M., Agagah G., Aedy H., Notter J. (2020). COVID-19 disease: Invasive ventilation. Clin. Integr. Care.

[171] Meng L., Qiu H., Wan L., Ai Y., Xue Z., Guo Q., Deshpande R., Zhang L., Meng J., Tong C. (2020). Intubation and Ventilation amid the COVID-19 Outbreak: Wuhan’s Experience. Anesthesiology.

[172] Alp E., Voss A. (2006). Ventilator associated pneumonia and infection control. Ann. Clin. Microbiol. Antimicrob..

[173] Chastre J., Fagon J. (2002). State of the Art Ventilator-associated Pneumonia. Am. J. Respir Crit Care Med..

[174] Kollef M.H. (2004). Prevention of hospital-associated pneumonia and ventilator-associated pneumonia. Crit. Care Med..

[175] Thakuria B., Singh P., Agrawal S., Asthana V. (2013). Profile of infective microorganisms causing ventilator-associated pneumonia: A clinical study from resource limited intensive care unit. J. Anaesthesiol. Clin. Pharmacol..

[176] Sarda C., Fazal F., Rello J. (2019). Management of ventilator-associated pneumonia (VAP) caused by resistant gram-negative bacteria: Which is the best strategy to treat?. Expert Rev. Respir. Med..

[177] Estes R.J., Meduri G.U. (1995). The pathogenesis of ventilator-associated pneumonia: I. Mechanisms of bacterial transcolonization and airway inoculation. Intensive Care Med..

[178] Bahrani-Mougeot F.K., Paster B.J., Coleman S., Barbuto S., Brennan M.T., Noll J., Kennedy T., Fox P.C., Lockhart P.B. (2007). Molecular analysis of oral and respiratory bacterial species associated with ventilator-associated pneumonia. J. Clin. Microbiol..

[179] Hayata M., Watanabe N., Tamura M., Kamio N., Tanaka H., Nodomi K., Miya C., Nakayama E., Ueda K., Ogata Y. (2019). The periodontopathic bacterium Fusobacterium nucleatum induced proinflammatory cytokine production by human respiratory epithelial cell lines and in the lower respiratory organs in mice. Cell. Physiol. Biochem..

[180] Shay K. (2002). Infectious complications of dental and periodontal diseases in the elderly population. Clin. Infect. Dis..

